# LncRNA OTUD6B-AS1 Induces Cisplatin Resistance in Cervical Cancer Cells Through Up-Regulating Cyclin D2 *via* miR-206

**DOI:** 10.3389/fonc.2021.777220

**Published:** 2021-10-22

**Authors:** Hui Hou, Rong Yu, Haiping Zhao, Hao Yang, Yuchong Hu, Yue Hu, Junmei Guo

**Affiliations:** ^1^ Department of Pediatric Hematology and Oncology, Inner Mongolia Autonomous Region People’s Hospital, Huhhot, China; ^2^ Department of Radiation Oncology, Inner Mongolia Cancer Hospital and Affiliated People’s Hospital of Inner Mongolia Medical University, Huhhot, China; ^3^ Department of Abdominal Tumor Surgery, Affiliated Hospital of Inner Mongolia Medical University, Huhhot, China; ^4^ Department of Gynaecology, Inner Mongolia Autonomous Region People’s Hospital, Huhhot, China

**Keywords:** miR-206, cisplatin resistance, cervical cancer, OTUD6B-AS1, CCND2

## Abstract

Cervical cancer is one of the most common gynecological cancers. Cisplatin resistance remains a major hurdle in the successful treatment of cervical cancer. Aberrant expression of long non-coding RNAs (lncRNAs) and microRNAs (miRNAs) are implicated in cisplatin resistance. However, the regulatory functions of lncRNAs and miRNAs in cervical cancer cisplatin resistance and the underlying mechanisms are still elusive. Our qRT-PCR assays verified that miR-206 levels were down-regulated in cisplatin-resistant cervical cancer cells. The introduction of miR-206 sensitized cisplatin-resistant cervical cancer cells to cisplatin. Our qRT-PCR and luciferase reporter assays showed that Cyclin D2 (*CCND2*) was the direct target for miR-206 in cervical cancer cells. The cisplatin-resistant cervical cancer cells expressed higher *CCND2* expression than the parental cells, whereas inhibition of CCND2 could sensitize the resistant cells to cisplatin treatment. Furthermore, we demonstrated that lncRNA OTUD6B-AS1 was up-regulated in cisplatin-resistant cervical cancer cells, and knocking down OTUD6B-AS1 expression induced re-acquirement of chemosensitivity to cisplatin in cervical cancer cells. We also showed that OTUD6B-AS1 up-regulated the expression of CCND2 by sponging miR-206. Low miR-206 and high OTUD6B-AS1 expression were associated with significantly poorer overall survival. Taken together, these results suggest that OTUD6B-AS1-mediated down-regulation of miR-206 increases CCND2 expression, leading to cisplatin resistance. Modulation of these molecules may be a therapeutic approach for cisplatin-resistant cervical cancer.

## Introduction

Cervical cancer is a common gynecological cancer (1). Cisplatin (CDDP)-based concurrent chemo-radiotherapy is the standard of care for cervical cancer patients (2). However, the intrinsic or acquired resistance to cisplatin often leads to the failure of cervical cancer treatment and disease progression (3). Therefore, elucidating the underlying mechanisms of CDDP resistance is crucial to improve cervical cancer patient survival.

Cyclin D2 (CCND2) is expressed in a broad range of tumor types, and it has key roles in the carcinogenesis and progression of cervical cancer (4–6). Previous studies have reported that CCND2 could influence CDDP resistance in bladder cancer (7, 8). Recently, CCND2 was shown to facilitate the malignant properties of cervical cancer cells (9). However, its functional roles in mediating CDDP resistance, and the mechanisms that control the expression of CCND2 in cervical cancer cells, remain poorly undefined.

MicroRNAs (miRNAs) modulate RNA degradation or translational repression through binding to the 3′-untranslated region (3′-UTR) of their target mRNAs (10). The expression of miR-206 is reported to be reduced in cervical cancer samples, and overexpression of miR-206 suppresses the growth, migration, and invasion of cervical cancer cells (11, 12). According to our bioinformatics analysis, *CCND2* was predicted to be a possible target gene of miR-206. However, the relationship between CCND2 and miR-206 in cervical cancer has not been clarified. Moreover, long non-coding RNAs (lncRNAs) are non-coding RNAs longer than 200 nucleotides that are involved in the regulation of various biological processes, including CDDP resistance (13). For instance, lncRNA NNT-AS1 expression was increased in CDDP-resistant cervical cancer samples and contributed to CDDP resistance in cervical cancer cells through the miR-186/HMGB1 axis (14). OTUD6B-AS1 is an antisense lncRNA located on chromosome 8:91059909-91070097, with three exons and a length of 4292 nucleotides. OTUD6B-AS1 acts as an important oncogenic lncRNA in renal cell carcinoma (15) and hepatocellular carcinoma (16). However, whether OTUD6B-AS1 could regulate CDDP resistance in cervical cancer cells by upregulating CCND2 expression through protecting it from miR-206-mediated repression is unknown.

Here, we found that the expression of miR-206 is reduced in CDDP-resistant cervical cancer cells. Importantly, forced expression of miR-206 could enhance the sensitivity of cervical cancer cells to CDDP through targeting oncogene *CCND2*. Furthermore, our results showed that lncRNA OTUD6B-AS1 promotes CDDP resistance in cervical cancer cells by up-regulating CCND2 expression *via* sequestering miR-206. Overall, our findings shed new insights on the mechanisms of CDDP resistance and purport possible clinical application of targeting the OTUD6B-AS1/miR-206/CCND2 pathway as a promising way to reverse CDDP resistance and improve the prognosis of patients with cervical cancer.

## Materials and Methods

### Patients and Tissue Samples

Thirty cancer samples and adjacent normal samples were collected from patients diagnosed with cervical cancer in the Affiliated Hospital of Inner Mongolia Medical University. Our research was approved by the Research Ethics Committee of Affiliated Hospital of Inner Mongolia Medical University. Each patient gave written informed consent before participating in this study. The acquired samples were stored at -80°C for the following research.

### Cell Culture and Cell Lines

We purchased human cervical cancer cell lines (HeLa, CaSki, and SiHa) and a normal ectocervical cell line (Ect1/E6E7) from the American Type Culture Collection (ATCC, Manassas, VA). These cells were maintained in DMEM/F12 medium (Sigma-Aldrich, St. Louis, MO) supplemented with 10% fetal bovine serum (FBS, Invitrogen, Carlsbad, CA).

### Generation of CDDP-Resistant Cervical Cancer Cells

CDDP-resistant cervical cancer cell lines were generated as previously described (17). In brief, CDDP-resistant cell lines were developed from their parental cells (HeLa and SiHa) using stepwise selection for resistance with increasing concentrations of CDDP. The resulting CDDP-resistant cell lines (HeLa-CDDP and SiHa-CDDP) were cultured in a CDDP-containing media to maintain the CDDP-resistant phenotypes.

### Cell Viability Assay

Cervical cancer cells (5 × 10^3^ cells per well) were seeded into 96-well plates and incubated for 24 h. Then, CDDP was diluted to different concentrations in DMEM/F12 medium and was added to the wells. The cells were incubated for an additional 24 h before CCK-8 solution (10 μl per well, Dojindo, Japan) was added to each well. The absorbance at 450 nm was subsequently measured using a microplate reader.

### Quantitative Real-Time PCR (qRT-PCR) Assay

Total RNA from cervical cancer samples or cervical cancer cell lines was isolated with TRIzol reagent (Invitrogen). The complementary DNA (cDNA) synthesis was performed using a PrimeScript reagent kit (TaKaRa, Beijing). Quantitative real-time PCR using SYBR Green (Takara) was conducted using the ABI-7300 Real-Time PCR system (Applied Biosystems, Foster City, CA). The following primers were used: VEGF-A-F: 5′-AGGGCAGAATCATCACGAAGT-3′ and VEGF-A-R: 5′-AGGGTCTCGATTGGATGGCA-3′; BAG3-F: 5′-TGGGAGATCAAGATCGACCC-3′ and BAG3-R: 5′-GGGCCATTGGCAGAGGATG-3′; EZH1-F: 5′-ATGCGACTTCGACAACTTAAACG-3′ and EZH1-R: 5′-GGCTTCATTGACTGAACAGGTT-3′; CCND2-F: 5′-ACCTTCCGCAGTGCTCCTA-3′ and CCND2-R: 5′-CCCAGCCAAGAAACGGTCC-3′; GAPDH-F: 5′-AATCCCATCACCATCTTC-3′ and GAPDH-R: 5′-AGGCTGTTGTCATACTTC-3′. The qRT-PCR analysis of OTUD6B-AS1 expression was performed using the following primers (OTUD6B-AS1-F: 5′-AAGCTACGCGCTAGGCTCTT-3′ and OTUD6B-AS1-R: 5′-GAGGTCCTCTGAAGCAGGGAA-3′) (18). The internal control for mRNA is given as the ratio to GAPDH. The levels of miR-206 were determined using the NCode SYBR GreenER miRNA qRT-PCR analysis kit (Invitrogen). The forward primer for miR-206 analysis had the same sequence as the mature miR-206. U6 (U6-F: 5′-GCTTCGGCAGCACATATACTAAAAT-3′ and U6-R: 5′-CGCTTCACGAATTTGCGTGTCAT-3′) served as an endogenous control.

### Cell Transfection

We transfected cervical cancer cells with miR-206 mimic, control mimic, miR-206 inhibitor, and control inhibitor using Lipofectamine 2000 reagent (Invitrogen). The siRNA targeting CCND2 or OTUD6B-AS1, as well as the control siRNA, were all obtained from Geneseed (Guangzhou) were transfected to cervical cancer cells using Lipofectamine 2000 (Invitrogen) according to the manufacturer’s protocol.

### Western Blotting Analysis

Total proteins were extracted using RIPA lysis buffer (FD, Hangzhou) containing a protease inhibitor cocktail on ice for 30 min. The equal amounts (30 µg) of protein were separated by 12% SDS-polyacrylamide gel electrophoresis and transferred to PVDF membranes (GE Healthcare Life Sciences, Piscataway, NJ). After blocking the membranes in 5% non-fat milk under room temperature for 1 h, primary antibodies (anti-CCND2 antibody and anti-GAPDH antibody (Abcam, Cambridge, MA) were added to the membrane and incubated overnight. Then, the membranes were subsequently incubated with the corresponding secondary antibodies and incubated at room temperature. The proteins were detected using an ECL detection kit (Amersham Pharmacia Biotech, UK).

### Luciferase Assay

Wild-type (WT) human CCND2 3′-UTR luciferase reporter vector was obtained from OriGene (Rockville, MD). Mutations (MUT) of the miR-206 binding site in the CCND2 3′-UTR sequence were created using the QuickChange site-directed mutagenesis kit (Stratagene, La Jolla, CA). The full-length of OTUD6B-AS1 was amplified and cloned into the pGL3-basic vector (Promega, Madison, WI). After cervical cancer cells were seeded onto 24-well plates, 100 ng of the luciferase reporter vector, 10 ng of the pRL-CMV vector (Promega, Madison, WI), as well as 30 nM of miR-206 mimic, control mimic, miR-206 inhibitor, or control inhibitor, were transfected to cancer cells using Lipofectamine 2000 (Invitrogen). At 48 h after transfection, luciferase activity was quantified using the Dual-Luciferase Reporter Assay System (Promega).

### Animal Experimental Protocol

The animal protocol was approved by the Animal Care and Use Committee of Affiliated Hospital of Inner Mongolia Medical University. The 4-week-old BALB/c nude mice were injected subcutaneously with 5 × 10^6^ CDDP-resistant SiHa cells. Treatment began at 8 days after injection of CDDP-resistant SiHa cells. Either 1 nmol of the control siRNA or 1 nmol of OTUD6B-AS1 siRNA was mixed with AteloGene Local Use Quick gelation (Koken, Tokyo, Japan). The mixture was subcutaneously injected around the tumor at days 12, 16, 20, and 24. Tumor volumes were calculated using the formula (L × W× W)/2, where L = length and W = width of the tumor. At the end of the experiment, mice were sacrificed and tumor weights were recorded.

### Statistical Analysis

Results represent the mean values ± standard deviations. Statistical analysis was conducted using SPSS 20.0 (SPSS, Chicago). Differences between groups were compared using Student’s t-test, Mann-Whitney U test, and one-way ANOVA test. The correlations were analyzed using the Pearson correlation coefficient. P-value < 0.05 was defined as statistical significance.

## Results

### MiR-206 Is Down-Regulated in CDDP-Resistant Cervical Cancer Cells

**Figure 1:**
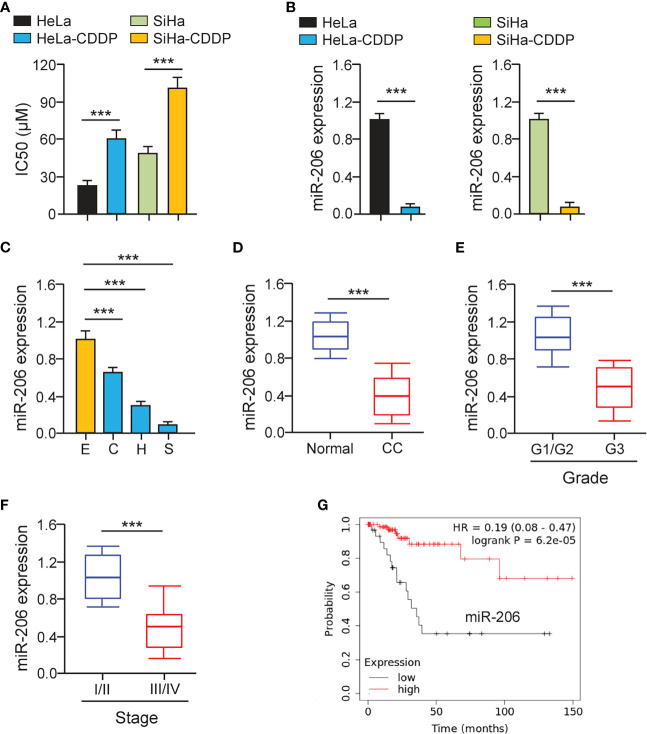
MiR-206 is Down-regulated in CDDP-resistant Cervical Cancer Cells. **(A)** Cell viability assay in response to CDDP was determined using CDDP-resistant HeLa (or SiHa) cells and their parent cells. **(B)** The qRT-PCR assays of miR-206 in CDDP-resistant cervical cancer cells and their parent cells. **(C)** The expression of miR-206 in SiHa (S), HeLa (H), CaSki (C) cells, and a normal ectocervical cell line Ect1/E6E7 (E) was analyzed with the qRT-PCR analysis. **(D)** The levels of miR-206 were examined in cervical cancer and adjacent normal samples using the qRT-PCR assays. **(E)** The expression of miR-206 in high-grade cervical cancer tissues and low-grade cervical cancer tissues. **(F)** The levels of miR-206 in cervical cancer samples with clinical early-stage or advanced-stage were investigated using qRT-PCR assays. **(G)** The overall survival of cervical cancer patients with higher or lower miR-206 expression was analyzed using the KM plotter database. ***P < 0.001.

First, we established CDDP-resistant HeLa and SiHa cells using repeated CDDP incubation. The half-maximal inhibitory concentration (IC50) values for CDDP were calculated using cell viability assays. CDDP-resistant HeLa and SiHa cells had much higher IC50 values for CDDP than the parental HeLa and SiHa cells (**Figure 1A**). CCND2 was previously predicted and confirmed to be a direct target gene of miR−206 in ovarian cancer cells (19). We speculated that miR-206 may increase the sensitivity of cervical cancer cells to CDDP by targeting CCND2. The qRT-PCR assays were performed to test the levels of miR-206 in CDDP-resistant HeLa (or SiHa) cells and the parent HeLa (or SiHa cells). We confirmed that the levels of miR-206 were remarkably decreased in CDDP-resistant HeLa (or SiHa) cells compared with that of the parent HeLa (or SiHa) cells (**Figure 1B**). Also, the expression of miR-206 was investigated in cervical cancer cell lines and Ect1/E6E7 cells using qRT-PCR analysis. Consistently, the levels of miR-206 were significantly reduced in cervical cancer cell lines (**Figure 1C**). Then, the qRT-PCR assays were used to examine its expression in 30 cervical cancer samples and adjacent normal samples. The expression of miR-206 was reduced drastically in cervical cancer samples compared to normal samples (**Figure 1D**). We further explored the correlation of miR-206 expression and clinical outcomes of cervical cancer patients. The qRT-PCR assay showed that lower expression of miR-206 was correlated with high-grade histology and the advanced stages of cervical cancer (**Figures 1E, F**). In a survival study, Kaplan-Meier survival analysis of cervical cancer patients from the KM plotter database (http://kmplot.com/analysis/) showed that the patients expressing lower miR-206 expression correlated with significantly shorter survival than those patients expressing high miR-206 expression (**Figure 1G**). The above results suggest that reduced expression of miR-206 might be correlated with CDDP resistance and poorer prognosis in cervical cancer.

### MiR-206 Mediates the Sensitivity of Cervical Cancer Cells to CDDP

**Figure 2:**
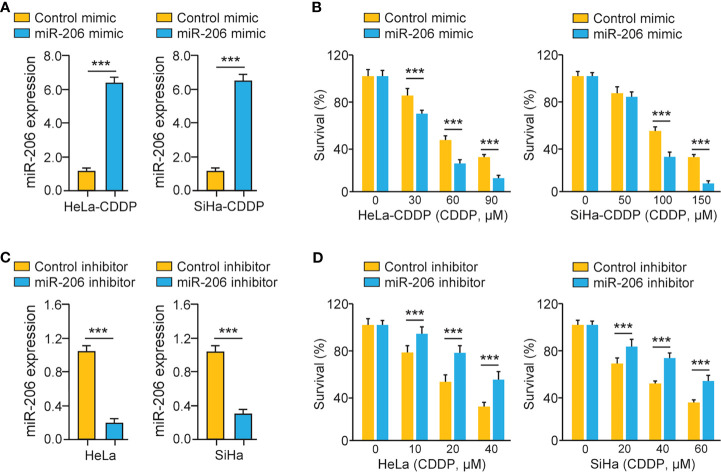
MiR-206 Mediates CDDP Resistance in Cervical Cancer Cells. **(A)** The qRT-PCR analysis of miR-206 in CDDP-resistant cervical cancer cells transfected with miR-206 mimic or control mimic. **(B)** After transfection with miR-206 mimic or control mimic, CDDP-resistant HeLa (or SiHa) cells were treated with CDDP and cell viability was measured using cell viability assays. **(C)** The qRT-PCR analysis of miR-206 in the parent cervical cancer cells transfected with miR-206 inhibitor or control inhibitor. **(D)** After transfection with miR-206 inhibitor or control inhibitor, the parent HeLa (or SiHa) cells were treated with CDDP and cell viability assays were conducted. ***P < 0.001.

We investigated whether miR-206 modulation could affect the chemosensitivity of CDDP-resistant cervical cancer cells *via* performing cell viability assays. CDDP-resistant HeLa (or SiHa) cells were transfected with miR-206 mimic (or control mimic) and treated with CDDP. A significant increase in miR-206 expression was induced by the miR-206 mimic (**Figure 2A**). Cell viability assays demonstrated that CDDP-resistant cervical cancer cells transfected with miR-206 mimic were more sensitive to CDDP compared with the corresponding control cells (**Figure 2B**). To verify these results, we transfected the parent HeLa and SiHa cells with miR-206 inhibitor (or control inhibitor) and treated these cells with CDDP. The transfection with miR-206 inhibitor led to the successful inhibition of miR-206 expression according to the qRT-PCR results (**Figure 2C**). The parental cervical cancer cells transfected with miR-206 inhibitor exhibited greater resistance to CDDP than the corresponding controls (**Figure 2D**). These results demonstrated that miR-206 modulates the sensitivity to CDDP in cervical cancer cells.

### *CCND2* Is a Target of MiR-206

**Figure 3:**
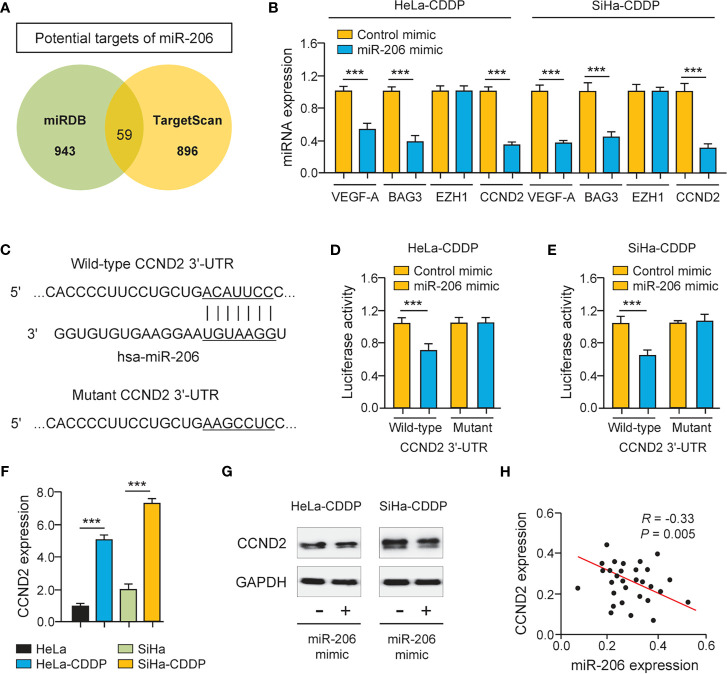
*CCND2* is a Target of MiR-206. **(A)** Venn diagram showing the overlapping genes predicted by two online databases (miRDB and TargetScan). **(B)** The qRT-PCR analysis of *BAG3*, *VEGF-A*, C*CND2*, and *EZH1* in CDDP-resistant HeLa (or SiHa) cells that were transfected with miR-206 mimic or control mimic. **(C)** Illustration of the predicted *CCND2* 3′-UTR-binding site for miR-206. **(D, E)** The CDDP-resistant HeLa **(D)** and SiHa **(E)** cells were transfected with miR-206 mimic (or control mimic) and luciferase vector containing the wild-type (WT) or mutant (MUT) *CCND2* 3′-UTR. After 48 h of incubation, the luciferase activity was determined. **(F)** The mRNA level of *CCND2* in CDDP-resistant HeLa (or SiHa) cells and their parent cells. **(G)** Western blotting analysis of CCND2 expression in CDDP-resistant HeLa (or SiHa) cells that were transfected with miR-206 mimic or control mimic. **(H)** The correlation between miR-206 and *CCND2* expression in 30 cervical cancer tissues. ***P < 0.001.

The miRNAs regulate the expression of their target genes, and two prediction databases (miRDB and TargetScan) were available for the prediction of the targets of miR-206. As a result, predicted target genes (including known miR-206 target gene BAG3 (11), VEGF-A (12), and CCND2) were identified (**Figure 3A**). To examine whether miR-206 regulates the expression of these putative genes, their mRNA levels were investigated using qRT-PCR assays. The expression of BAG3, VEGF-A, and CCND2 showed a significant decrease after transfection with miR-206 mimic in CDDP-resistant HeLa and SiHa cells, whereas the level of an unrelated gene EZH1 was not significantly altered (**Figure 3B**). We used luciferase assays to determine the possibility that miR-206 could directly repress the expression of CCND2 in CDDP-resistant HeLa and SiHa cells. The luciferase activity of the reporter vector containing the wild-type CCND2 3′-UTR was inhibited by miR-206 mimic, whereas this miR-206 mimic did not affect the luciferase activity of the reporter construct containing the mutant CCND2 3′-UTR (**Figures 3C-E**). Furthermore, the mRNA expression of CCND2 was higher in the CDDP-resistant HeLa (or SiHa) cells than in their parent cells (**Figure 3F**). The transfection with miR-206 mimic reduced the protein level of CCND2 in the CDDP-resistant HeLa (or SiHa) cells (**Figure 3G**). In cervical cancer tissues, the expression of miR-206 and CCND2 was inversely correlated (**Figure 3H**). These results suggest that CCND2 is a direct target of miR-206 in cervical cancer cells and CCND2 expression is increased during the acquisition of CDDP resistance.

### Up-Regulation of CCND2 Induces CDDP Resistance in Cervical Cancer Cells

**Figure 4:**
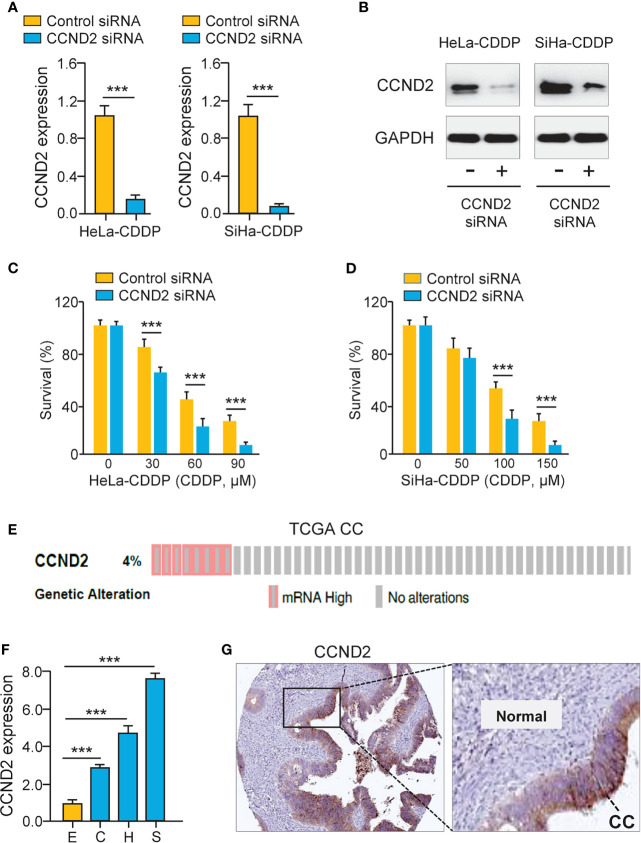
Up-regulation of CCND2 Induces CDDP resistance in Cervical Cancer Cells. **(A)** The qRT-PCR analysis of C*CND2* expression in CDDP-resistant HeLa (or SiHa) cells that were transfected with CCND2 siRNA or control siRNA. **(B)** Western blotting analysis of C*CND2* expression in CDDP-resistant HeLa (or SiHa) cells that were transfected with CCND2 siRNA or control siRNA. **(C, D)** After transfection with CCND2 siRNA or control siRNA, the CDDP-resistant HeLa **(C)** and SiHa **(D)** cells were treated with CDDP and cell viability assays were performed. **(E)** The frequency of CCND2 genomic changes in the TCGA cervical cancer tissues (cBioPortal database). **(F)** The mRNA expression of CCND2 in SiHa (S), HeLa (H), CaSki (C) cells, and a normal ectocervical cell line Ect1/E6E7 (E) was analyzed with the qRT-PCR analysis. **(G)** The protein expression of CCND2 was examined in cervical cancer samples (Human Protein Atlas database). CC, Cervical cancer. ***P < 0.001.

To test the functional impact of CCND2 on cervical cancer cell chemosensitivity, the siRNA specific to CCND2 was transfected to CDDP-resistant HeLa and SiHa cells. The qRT-PCR assay and western blotting analysis confirmed the down-regulation of CCND2 in CDDP-resistant cells transfected with CCND2 siRNA (**Figures 4A, B**). Cell viability assays showed that the CDDP-resistant HeLa (or SiHa) cells that were transfected with CCND2 siRNA were more sensitive to CDDP treatment compared with the corresponding control cells (**Figures 4C, D**). Then, we evaluated the expression of CCND2 in the TCGA cervical cancer tissues using the cBioPortal database (https://www.cbioportal.org/) and found that CCND2 was frequently overexpressed in cervical cancer tissues (**Figure 4E**). Using the qRT-PCR assay, we detected higher CCND2 expression in cervical cancer cells than normal Ect1/E6E7 cells (**Figure 4F**). We analyzed the CCND2-stained IHC images obtained from the Human Protein Atlas database (https://www.proteinatlas.org/). Significant up-regulation of CCND2 was observed in cervical cancer samples when compared with adjacent normal samples (**Figure 4G**). Therefore, CCND2 is overexpressed in cervical cancer tissues and increases cervical cancer cell viability after CDDP exposure.

### LncRNA OTUD6B-AS1 Is Up-Regulated in CDDP-Resistant Cervical Cancer Cells

**Figure 5:**
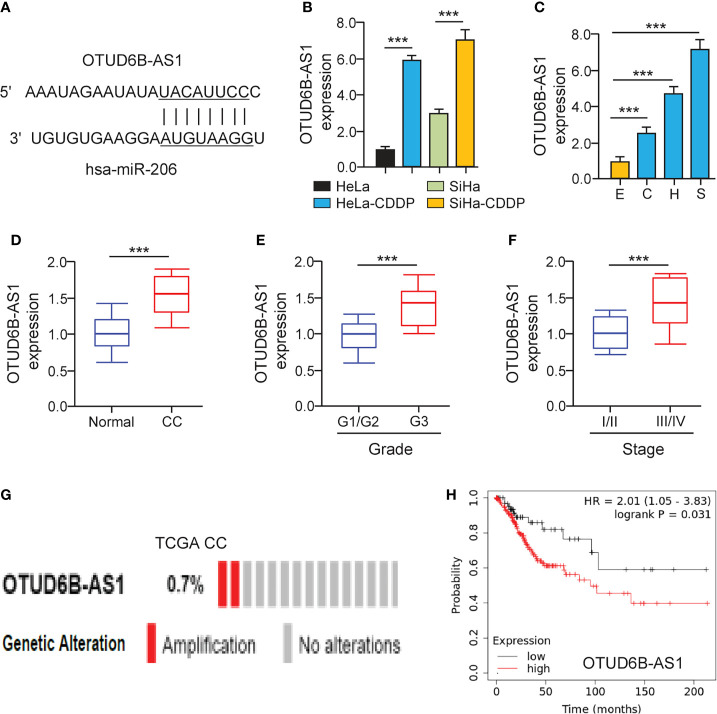
LncRNA OTUD6B-AS1 is Up-regulated in CDDP-resistant Cervical Cancer Cells. **(A)** The predicted miR-206 binding site in lncRNA OTUD6B-AS1. **(B)** The qRT-PCR analysis of OTUD6B-AS1 in CDDP-resistant cervical cancer cells and their parent cells. **(C)** The expression of OTUD6B-AS1 in SiHa (S), HeLa (H), CaSki (C) cells, and a normal ectocervical cell line Ect1/E6E7 (E) was examined using the qRT-PCR analysis. **(D)** The qRT-PCR analysis of OTUD6B-AS1 expression in cervical cancer and normal samples. **(E)** OTUD6B-AS1 expression in high-grade and low-grade cervical cancer samples. **(F)** OTUD6B-AS1 expression in advanced-stage and early-stage cervical cancer samples. **(G)** The frequency of OTUD6B-AS1 genetic changes in the TCGA cervical cancer tissues (cBioPortal database). **(H)** The overall survival of cervical cancer patients with higher or lower OTUD6B-AS1 expression was analyzed using the KM plotter database. ***P < 0.001.

It has been suggested that lncRNAs can bind with miRNAs to regulate the expression of downstream genes of miRNAs (20). To find miR-206-interacting lncRNAs, we used the ENCORI database (http://starbase.sysu.edu.cn/) to predict the possible binding between miR-206 and lncRNAs. We showed that there was a putative binding site of miR-206 in lncRNA OTUD6B-AS1 (**Figure 5A**). Using the qRT-PCR assay, we validated that the expression of OTUD6B-AS1 was elevated in CDDP-resistant cervical cancer cells (**Figure 5B**). Additionally, OTUD6B-AS1 expression was up-regulated in cervical cancer cell lines than normal cells (**Figure 5C**). In line with these results, OTUD6B-AS1 expression was induced in cervical cancer samples than normal tissues (**Figure 5D**). Also, the levels of OTUD6B-AS1 were significantly induced in high-grade cervical cancer tissues (**Figure 5E**) and advanced cervical cancer tissues (**Figure 5F**). We retrieved the TCGA cervical cancer datasets from cBioPortal and evaluated OTUD6B-AS1 alterations. OTUD6B-AS1 displayed frequent amplification over cervical cancer tissues (**Figure 5G**). According to the results from the KM plotter database, we observed that cervical cancer patients with higher levels of OTUD6B-AS1 have poorer overall survival (**Figure 5H**). These results suggest that induced expression of OTUD6B-AS1 is linked to CDDP resistance and poor clinical outcomes in cervical cancer.

### LncRNA OTUD6B-AS1 Interacts With miR-206 in CDDP-Resistant Cervical Cancer Cells

**Figure 6:**
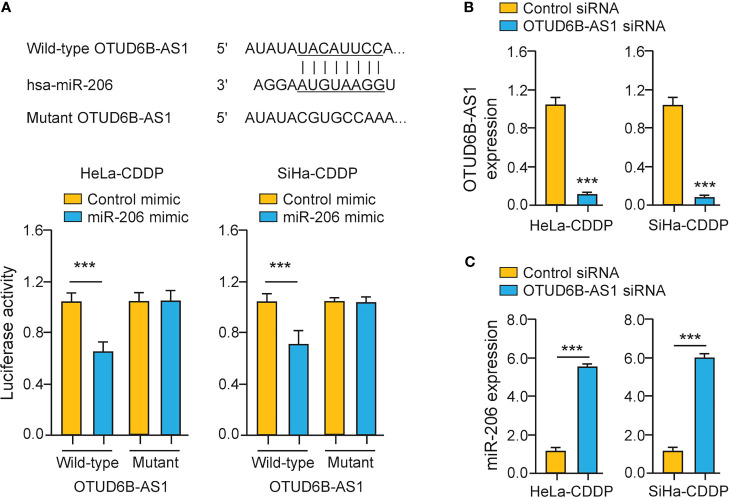
LncRNA OTUD6B-AS1 Interacts with MiR-206 in CDDP-Resistant Cervical Cancer Cells. **(A)** Upper: putative and mutant miR-206-binding sites in OTUD6B-AS1; lower: Luciferase reporter assays were performed in CDDP-resistant cervical cancer cells that were transfected with the wild-type (WT) or mutant (MUT) OTUD6B-AS1, with miR-206 mimic or control mimic. **(B, C)** The expression of OTUD6B-AS1 **(B)** miR-206 **(C)** in CDDP-resistant cervical cancer cells transfected with OTUD6B-AS1 siRNA or control siRNA. ***P < 0.001.

To explore whether miR-206 directly binds to OTUD6B-AS1, we performed luciferase assays in CDDP-resistant cervical cancer cells. The overexpression of miR-206 significantly suppressed the luciferase activity of the wild-type OTUD6B-AS1 fragment, while the miR-206 mimic had no significant effect on mutant OTUD6B-AS1 fragment (**Figure 6A**), suggesting that OTUD6B-AS1 is a direct target of miR-206. In CDDP-resistant cervical cancer cells, silencing of OTUD6B-AS1 significantly increased the levels of miR-206 (**Figures 6B, C**). Together, lncRNA OTUD6B-AS1 negatively regulates the expression of miR-206 through direct interaction.

### LncRNA OTUD6B-AS1 Induces CCND2 Expression and Promotes CDDP Resistance in Cervical Cancer Cells

**Figure 7:**
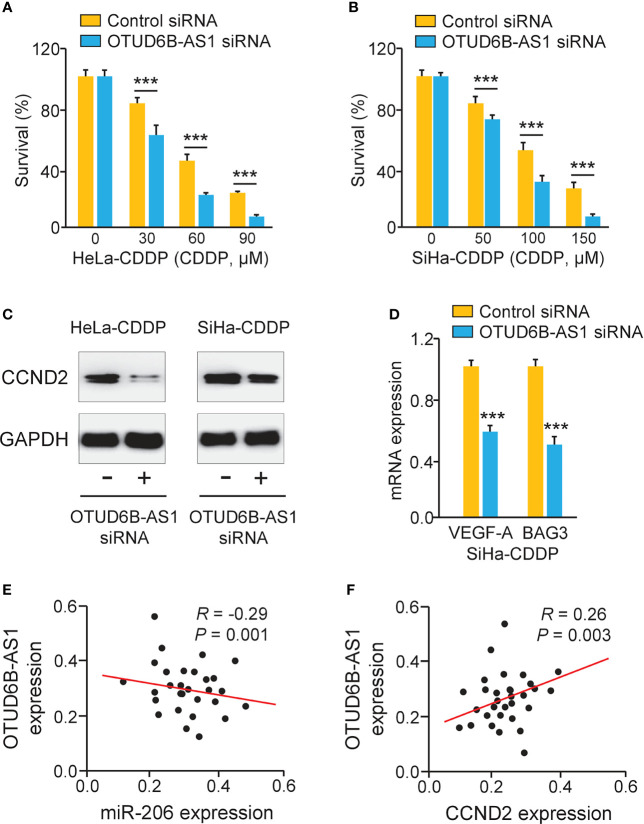
LncRNA OTUD6B-AS1 Induces CCND2 Levels and Promotes CDDP resistance in Cervical Cancer Cells. **(A, B)** After transfection with OTUD6B-AS1 siRNA or control siRNA, the CDDP-resistant HeLa **(A)** and SiHa **(B)** cells were treated with CDDP and cell viability assays were performed. **(C)** Western blotting analysis of C*CND2* expression in CDDP-resistant HeLa (or SiHa) cells that were transfected with OTUD6B-AS1 siRNA or control siRNA. **(D)** The qRT-PCR analysis of *BAG3* and *VEGF-A* in CDDP-resistant SiHa cells transfected with OTUD6B-AS1 siRNA or control siRNA. **(E, F)** The correlation between OTUD6B-AS1 and miR-206 expression and between OTUD6B-AS1 and *CCND2* expression in cervical cancer samples was analyzed. ***P < 0.001.

Because OTUD6B-AS1 is potentially implicated in the regulation of CDDP resistance, we investigated whether OTUD6B-AS1 could modulate CDDP resistance through increasing CCND2 expression. Knockdown of OTUD6B-AS1 in CDDP-resistant HeLa and SiHa cells significantly increased the sensitivity of cervical cancer cells to CDDP treatment (**Figures 7A, B**). We also observed that OTUD6B-AS1 knockdown markedly decreased the expression of CCND2, VEGF-A, and BAG3 in CDDP-resistant HeLa and SiHa cells (**Figures 7C, D**). In cervical cancer tissues, the expression of OTUD6B-AS1 was negatively correlated with the expression of miR-206, but was positively associated with the level of CCND2 (**Figures 7E, F**). These results supported the hypothesis that highly expressed OTUD6B-AS1 induces CDDP resistance in cervical cancer cells by increasing CCND2 levels *via* sponging miR-206.

### Silencing of lncRNA OTUD6B-AS1 Reduces the Growth of CDDP-Resistant Cervical Cancer Cells *In Vivo*

**Figure 8:**
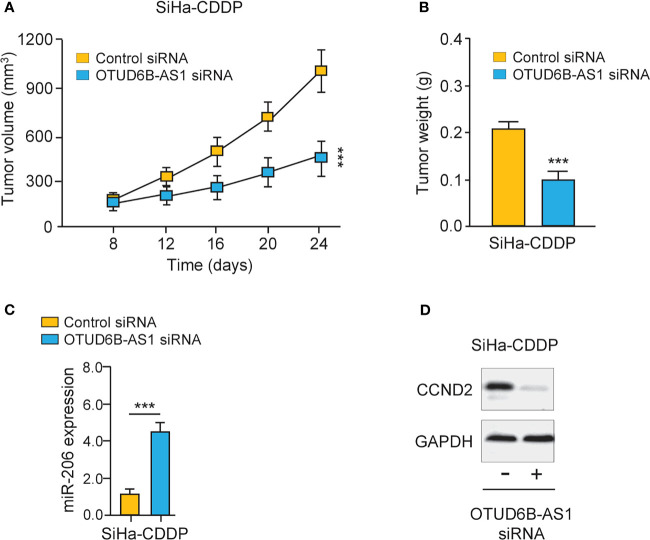
LncRNA OTUD6B-AS1 promotes the progression of CDDP-resistant cervical cancer cells *in vivo.* **(A, B)** The subcutaneous injection of the OTUD6B-AS1 siRNA significantly suppressed the growth of CDDP-resistant SiHa cells. Tumor growth **(A)** and tumor weights **(B)** of mice at 24 days were shown. **(C, D)** miR-206 **(C)** and CCND2 **(D)** levels were analyzed by qRT-PCR and western blotting assays in SiHa-CDDP-control siRNA and SiHa-CDDP-OTUD6B-AS1siRNA xenografts, respectively. ***P < 0.001.

Finally, we carried out animal experiments to investigate the function of OTUD6B-AS1 *in vivo*. Eight days after the subcutaneous injection of CDDP-resistant SiHa cells, the presence of tumors in the injection sites was visually confirmed. OTUD6B-AS1 siRNA or control siRNA with atelocollagen was subcutaneously injected around the tumor at days 12, 16, 20, and 24. Compared with control siRNA, OTUD6B-AS1 siRNA significantly attenuated the growth of CDDP-resistant SiHa cells *in vivo* (**Figures 8A, B**). Furthermore, we have detected the levels of miR-206 and CCND2 in xenografts. The qRT-PCR and western blotting assays have shown that miR-206 expression was induced, while CCND2 levels were reduced in OTUD6B-AS1 siRNA-treated group compared with the control group (**Figures 8C, D**). These findings suggest that OTUD6B-AS1 could promote the growth of CDDP-resistant cervical cancer cells *in vivo*.

## Discussion

CDDP is a commonly used anti-tumor drug for the treatment of cervical cancer (21). However, reversing intrinsic and acquired chemoresistance to CDDP remains a major challenge in treating cervical cancer patients (3). In this study, we investigated the mechanisms of CDDP resistance, which are crucial for improving the survival rates of cervical cancer patients.

CDDP resistance may develop as a result of the reduced import of CDDP, increased efflux of CDDP, increased DNA damage repair, inactivation of cell apoptosis, certain transcription factors, induction of epithelial-to-mesenchymal transition (EMT), DNA methylation, dysregulated miRNA expression, and gain of cancer stem cells (3). Recent discoveries of miRNAs and lncRNAs have provided a new direction for researchers to explore the mechanism of CDDP resistance. Both miRNAs and lncRNAs are closely involved in this process (22, 23). For instance, miR-206 was shown to be down-regulated in CDDP-resistant lung cancer cells, and overexpression of miR-206 reversed the mesenchymal features and sensitized CDDP-resistant cells to CDDP *via* targeting MET (24). In addition, up-regulation of miR-206 can decrease DDP resistance in gastric cancer cells *via* the inhibition of MAPK3 expression (25). In this study, we generated CDDP-resistant cervical cancer cell lines *via* continuous exposure to CDDP. By analyzing the expression of miR-206 in CDDP-resistant cervical cancer cells and their parent cells *via* qRT-PCR analysis, we revealed that miR-206 expression was dramatically down-regulated during the acquisition of CDDP resistance, and found that restoration of miR-206 led to the sensitization of CDDP-resistant cells to CDDP. Hence, our findings suggested that reduced expression of miR-206 is associated with CDDP resistance, and miR-206 expression may be a potential predictive biomarker for CDDP-based therapy efficacy in cervical cancer patients.

Previous studies have shown that the levels of miR-206 were diminished in cervical cancer when compared with normal tissues (26, 27). Lower levels of miR-206 in cervical cancer patients were associated with poor differentiation, advanced FIGO stage, positive lymph node metastasis, and human papillomavirus infection (27). Kaplan-Meier survival analysis further revealed that the down-regulation of miR-206 was strongly related to shorter overall survival in cervical cancer patients (26). Their results were completely consistent with our data showing that lower miR-206 expression was correlated with high-grade histology, the advanced stages of cervical cancer, and worse overall survival. Our data suggested that miR-206 is a valuable prognostic indicator of patients with cervical cancer.

Remarkably, our results have identified CCND2 as a direct miR-206 target in cervical cancer cells. CCND2 is a well-established oncogene that is commonly overexpressed in human tumors (4–6). Evidence has shown that elevated expression of CCND2 contributed to CDDP resistance in bladder cancer cells (7, 8). Although CCND2 could increase the proliferation and migration of cervical cancer cells (9), it remains unclear whether CCND2 mediates CDDP resistance in cervical cancer cells. Here, we showed for the time that suppression of CCND2 decreased CDDP resistance in cervical cancer cells. Previous studies have provided evidence that miR-195 (28) and miR-607 (9) are responsible for the regulation of CCND2 in CC. Interestingly, a previous study detected a negative regulation relationship between miR-206 and CCND2 in ovarian cancer (19). This study is the first to report that CCND2 was a direct downstream target of miR-206 in CDDP-resistant cervical cancer cells. Except for CCND2, many genes are likely to be regulated by miR-206. For instance, BAG3 and VEGF-A induced the proliferation and invasion of cervical cancer cells, and BAG3 and VEGF-A were direct target genes of miR-206 in cervical cancer (11, 12). Of note, down-regulation of BAG3 blocked CDDP−induced autophagy, thereby increasing CDDP sensitivity in ovarian cancer cells (29). We discovered that miR-206 could inhibit the expression of both BAG3 and VEGF-A in CDDP-resistant cervical cancer cells. Collectively, our results suggested that miR-206 acts as an important tumor suppressor that represses CDDP resistance by affecting the level of CCND2, BAG3, VEGF-A, and other downstream genes of miR-206.

Abundant researches have investigated the association of lncRNAs and drug resistance in cervical cancer. LncRNA NNT-AS1 contributed to CDDP resistance in cervical cancer by the miR-186/HMGB1 pathway (14). Another study indicated that lncRNA HNF1A-AS1 up-regulated the expression of TUFT1 by competing with miR-34b, thus reducing the sensitivity of cervical cancer cells to CDDP (30). Some studies have reported that lncRNA OTUD6B-AS1 functioned as an oncogenic lncRNA in the proliferation and invasion of renal cell carcinoma (15) and hepatocellular carcinoma cells (16). However, the relationship between OTUD6B-AS1 and CDDP resistance in cervical cancer, as well as the underlying mechanisms, remains unknown. Our research demonstrated for the first time that knocking down OTUD6B-AS1 could induce re-sensitization in the CDDP-resistant cervical cancer cells, and further revealed that OTUD6B-AS1 played its role through sponging miR-206. We have detected that OTUD6B-AS1 expression was much higher in CDDP-resistant cervical cancer cells than the parent cells, suggesting the possibility that a high level of OTUD6B-AS1 might be a predictive biomarker for CDDP resistance, and a potential therapeutic target for cervical cancer.

Increasing evidence has shown that the interactions between tumor cells and the tumor microenvironment can produce a niche that promotes drug resistance (31). Tumor angiogenesis is an important factor in determining the effectiveness of anti-cancer drugs (32). VEGF-A induces pathological angiogenesis and facilitates the self-renewal potentials of cancer stem cells (33), eventually contributing to tumor progression and therapy resistance (34). Our findings have indicated a possible mechanism between lncRNA OTUD6B-AS1 and VEGF-A: OTUD6B-AS1 suppresses the expression of miR-206 and elevates the levels of VEGF-A, resulting in CDDP resistance in cervical cancer. Continued work will be warranted to understand the relationship between the OTUD6B-AS1/miR-206 pathway and VEGF-A-associated angiogenesis and cancer stemness in cervical cancer.

## Conclusion

**Figure 9:**
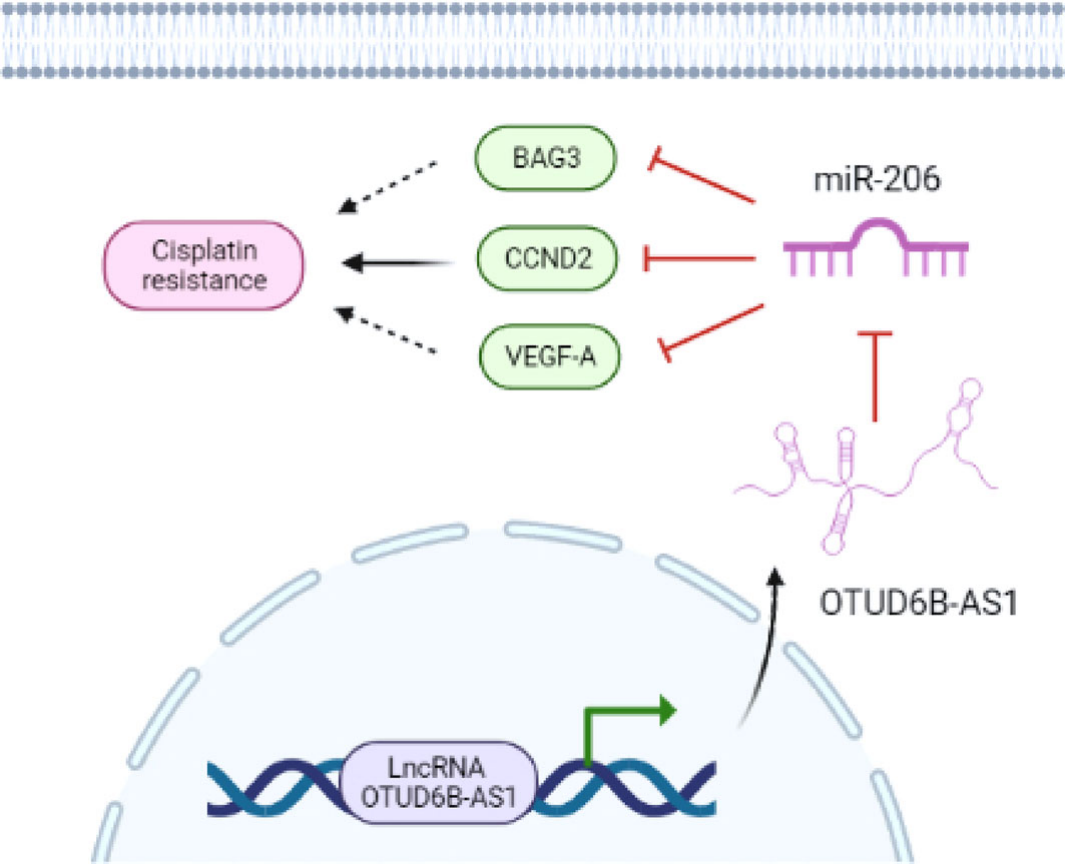
The model underlying the role of OTUD6B-AS1/miR-206/CCND2 axis in CC CDDP resistance. LncRNA OTUD6B-AS1 functions as a tumor promoter by sponging miR-206, weakening the effects of miR-206 on CCND2 and enhancing CDDP resistance in cervical cancer cells. Images were created with BioRender.com.

Our research showed that down-regulation of miR-206 led to the acquisition of CDDP resistance in cervical cancer cells, and the restoration of miR-206 attenuated CDDP resistance through targeting CCND2. LncRNA OTUD6B-AS1-induced suppression of miR-206 resulted in up-regulation of CCND2 and CDDP resistance (**Figure 9**). Thus, our findings reveal that OTUD6B-AS1, miR-206, and CCND2 may be useful therapeutic targets for overcoming CDDP resistance in patients with cervical cancer.

## Data Availability Statement

The original contributions presented in the study are included in the article/supplementary material. Further inquiries can be directed to the corresponding authors.

## Ethics Statement

The studies involving human participants were reviewed and approved by Research Ethics Committee of Affiliated Hospital of Inner Mongolia Medical University, China. The patients/participants provided their written informed consent to participate in this study. The animal study was reviewed and approved by Animal Care and Use Committee of Affiliated Hospital of Inner Mongolia Medical University, China.

## Author Contributions

HZ and HY designed the experiments. HH and RY performed the experiments. YCH, YH, and JG analyzed the data. All authors contributed to the article and approved the submitted version.

## Funding

This work was supported by a grant from the Natural Science Foundation of China (81860534), Inner Mongolia autonomous region science and technology planning project (2019GG039, 2019GG086, 2021GG0167), Xisike-Shiyao Clinical Oncology Research Foundation (Y-SY201901-0008), and Xisike-Qilu Clinical Oncology Research Foundation (Y-QL2019-0137).

## Conflict of Interest

The authors declare that the research was conducted in the absence of any commercial or financial relationships that could be construed as a potential conflict of interest.

## Publisher’s Note

All claims expressed in this article are solely those of the authors and do not necessarily represent those of their affiliated organizations, or those of the publisher, the editors and the reviewers. Any product that may be evaluated in this article, or claim that may be made by its manufacturer, is not guaranteed or endorsed by the publisher.
